# Tumor-derived extracellular vesicles shuttle c-Myc to promote gastric cancer growth and metastasis via the KCNQ1OT1/miR-556-3p/CLIC1 axis

**DOI:** 10.1038/s41419-021-04446-5

**Published:** 2022-03-08

**Authors:** Bopei Li, Yeyang Chen, Liang Liang, Ye Wang, Weijia Huang, Kun Zhao, Siyu Liu, Guofei Deng, Junqiang Chen

**Affiliations:** 1 Departments of Gastrointestinal Surgery, The First Affiliated Hospital of Guangxi Medical University, 530021 Nanning, P.R. China; 2 Guangxi Key Laboratory of Enhanced Recovery after Surgery for Gastrointestinal Cancer, 530021 Nanning, P.R. China; 3 Departments of Gastrointestinal Surgery, The First People’s Hospital of Yulin, 537000 Yulin, P.R. China; 4 Department of General Surgery, The Second Affiliated Hospital of Guangxi Medical University, 530007 Nanning, P.R. China; 5 Departments of Hepatobiliary Surgery, The First Affiliated Hospital of Guangxi Medical University, 530021 Nanning, P.R. China

**Keywords:** Cancer, Biotechnology

## Abstract

Gastric cancer (GC) is a heterogeneous disease with poor prognosis. Tumor-derived extracellular vesicles (EVs) assume a role in intercellular communication by carrying various molecules, including proteins, RNA, and DNAs, which has been identified to exhibit oncogenic effect in GC. Therefore, this research aimed to figure out whether tumor-derived EVs transmit c-Myc to orchestrate the growth and metastasis of GC. KCNQ1OT1, microRNA (miR)-556-3p and CLIC1 expression of GC tissues was detected through RT-qPCR. EVs were isolated from GC cells, followed by RT-qPCR and Western blot analysis of c-Myc expression in EVs and GC cells. Next, GC cells were incubated with EVs or transfected with a series of mimic, inhibitor, or siRNAs to assess their effects on cell viability, migrative, invasive, and apoptotic potential. Relationship among c-Myc, KCNQ1OT1, miR-556-3p, and CLIC1 was evaluated by dual-luciferase reporter assay. PI3K/AKT pathway-related proteins were assessed through Western blot analysis. KCNQ1OT1 and CLIC1 were highly expressed but miR-556-3p in GC tissues. c-Myc was high-expressed in tumor-derived EVs and GC cells. Mechanistically, c-Myc could induce KCNQ1OT1 expression, and KCNQ1OT1 bound to miR-556-3p that negatively targeted CLIC1 to inactivate PI3K/AKT pathway. Tumor-derived EVs, EVs-c-Myc, KCNQ1OT1 or CLIC1 overexpression, or miR-556-3p inhibition promoted GC cell proliferative, invasive, and migrative capacities but repressed their apoptosis through activating PI3K/AKT pathway. Collectively, tumor-derived EVs carrying c-Myc activated KCNQ1OT1 to downregulate miR-556-3p, thus elevating CLIC1 expression to activate the PI3K/AKT pathway, which facilitated the growth and metastasis of GC.

## Introduction

As the Global Cancer Statistics reported, there are 18.1 million newly diagnosed cancer cases and 9.6 million cancer-related deaths in 2018, of which gastric cancer (GC) accounts for 5.5% and 8.3%, respectively [1]. Intriguingly, GC incidence exhibits a wide geographical distribution with the highest rates observed in Southern America, Eastern Europe, and Eastern Asia [2]. In addition, due to the diversity of histopathological classifications, GC is a very heterogeneous disease from the morphologic standpoint [3]. Importantly, the 5-year survival rate of GC depends largely on the clinical stag with the range of 10-93%, and the survival rate of Asian patients is higher than that of Western patients [4]. Unfortunately, there exist limited treatment options in both locally advanced and metastatic GC, which contributes to poor prognosis [5]. Therefore, it is necessary to search more effective and safe therapies for GC.

As reported, various types of tumor cells can release extracellular vesicles (EVs) to participate in the orchestration of both physiological and pathological processes [6]. Moreover, tumor-derived EVs carry carries a series of biological molecules, like carbohydrates, lipids, nucleic acids, and proteins, and transports their cargo between adjacent cells as well as distant cells, thus acting as a messenger of intercellular communication [7]. It has been documented that tumor-derived EVs are capable of inducing progression and metastasis of GC [8]. More importantly, tumor-derived EVs could shuttle c-Myc to elevate lung bronchial cell proliferation, thus promoting lung cancer development [9]. Interestingly, it was indicated in a prior work that c-Myc increased GC cell growth to promote GC development [10]. Notably, another research uncovered that c-Myc could augment long noncoding RNA (lncRNA) potassium voltage-gated channel subfamily Q member 1 overlapping transcript 1 (KCNQ1OT1) expression in hepatic stellate cells [11]. However, there exists limited research about the manipulation of KCNQ1OT1 by c-Myc in tumors, which warrants further studies. Additionally, KCNQ1OT1 is able to accelerate GC progression by enhancing cell growth [12].

Furthermore, LncBase used in our study predicted that there exist binding sites of KCNQ1OT1 to microRNA (miR)-556-3p. miR-556-3p is elucidated as a tumor suppressor in tumorigenesis. For instance, miR-556-3p upregulation resulted in decrease of lung cancer cell viability, migration, and invasion [13]. Besides, it was presumed by TargetScan website in our work that miR-556-3p had the binding site to chloride intracellular channel protein 1 (CLIC1). Ectopic expression of CLIC1 could activate cancer-associated fibroblasts to facilitate GC cell proliferation and migration [14]. Based on this, this research was conducted to figure out whether tumor-derived EVs delivered c-Myc to manipulate KCNQ1OT1/miR-556-3p/CLIC1 axis to affect GC development.

## Materials and methods

### Ethics statement

All participants or their guardians provided signed informed consent before enrollment. The Ethics Committee of The First Affiliated Hospital of Guangxi Medical University provided Ethical Approval for human experiments that conformed to the *Declaration of Helsinki*.

### Bioinformatics analysis

The differentially expressed genes (DEGs) in GC were analyzed through bioinformatics online tool Gene Expression Profiling Interactive Analysis (GEPIA) with |logFC | > 1, *p* value < 0.05 as the screening criteria. The key word “GC” was searched in GeneCards database to retrieve the genes related to GC. hTFtargets which was a bioinformatics prediction website was utilized to predict the targeting factors of transcription factors and obtain the possible binding sites between the transcription factor and the lncRNA (Supplementary Fig. 1). The overlapping parts of ncRNA related to GC and candidate targeting factors in hTFtargets were found by jvenn online tool. The miRNAs binding to the lncRNA were predicted by the LncBase database. miRNAs related to GC were presumed by MNDR v3.1 and MISIM v2.0. Jvenn online tool was adopted to find the overlap of candidate miRNA of the lncRNA and miRNAs related to GC. The expression of the miRNA in GC samples was analyzed by dbDEMC 2.0 website (http://www.picb.ac.cn/dbDEMC/). To predict the downstream orchestrating factors of the miRNA, TargetScan and miRDB were employed to predict the downstream target genes of the miRNA, and jvenn tool was applied to find the overlaps of target genes, GC-related genes and upregulated genes. We analyzed the Kyoto Encyclopedia of Genes and Genomes (KEGG) pathways involved by genes through the Network Analyst website (https://www.networkanalyst.ca/) to further obtain the related pathways of target genes.

### Clinical samples

Forty GC and adjacent normal tissues were attained from primary GC patients. All patients had not undergone chemotherapy or radiotherapy. The tissues were harvested during gastroscopy and immediately preserved at −80 °C.

### Cell incubation and transfection

Incubation of GC cell lines SNU-1 (CC-Y1478) and NCI-N87 (CC-Y1398) (EK-Bioscience, Shanghai, China; http://www.elisakits.cn/Index/product/ccid/147.html) was conducted in RPMI 1640 complete medium (12633012; Gibco, Invitrogen, USA; https://www.thermofisher.cn/) encompassing 10% fetal bovine serum (FBS; 10099141; Gibco) and 1% penicillin-streptomycin (15070063; Gibco). Incubation of HEK-293T (CC-Y1010; EK-Bioscience) was conducted in Dulbecco’s modified Eagle’s medium (DMEM) (10569044; Gibco) encompassing 10% FBS and 1% penicillin-streptomycin. GC cells were cultured in a 37 °C incubator with 5% CO_2_.

The cells were transfected with small interfering RNA (si)-negative control (NC), si-c-Myc#1, or si-c-Myc#2; transfected with si-NC, si-c-Myc, or si-c-Myc + overexpression (oe)-KCNQ1OT; transfected with si-NC, si-KCNQ1OT1, or si-KCNQ1OT1 + miR-556-3p inhibitor; or transfected with miR-NC, miR-556-3p mimic, inhibitor NC, miR-556-3p inhibitor, oe-NC, oe-CLIC1, oe-CLIC1 + dimethyl sulfoxide (DMSO), or oe-CLIC1 + LY294002.

The cell models of overexpressing NC and overexpressing CLIC1 were constructed in GC cells which named oe-NC and oe-CLIC1 respectively. GC cells were incubated in a 6-well plate with 2 mL ligand per well. 800 μL fresh virus solution was mixed with 800 μL FBS, followed by supplementing of Polybrene until the final concentration was 6 μg/mL. After 12–24 h of culture, the medium was changed to complete medium to culture sequentially at 37 °C with 5% CO_2_. After infection for 48 h, the cells were cultured in puromycin (1 μg/mL) medium for 2 weeks to screen stable transfected cell lines. When the cells did not dead in puromycin-encompassing medium, the cell protein was extracted. Then the overexpression of genes or proteins was confirmed by reverse transcription quantitative polymerase chain reaction (RT-qPCR) or Western blot analysis.

Cells were seeded into a 6-well plate and transfected with miR-556-3p mimic, miR-NC, miR-556-3p inhibitor, or inhibitor NC (50 nM, GenePharma, Shanghai, China) by Lipofactamine 2000 reagent (Invitrogen, Carlsbad, California, USA). Then the packaging mixture was transfected into GC cells. si-NC, si-c-Myc, or si-KCNQ1OT1 (30 nmol/L; Invitrogen) were transfected into GC cells (Each gene had at least two siRNA interference sequences, and the siRNA sequence with the best knockdown efficiency was chosen for the following experiments) by Lipofactamine 2000 reagent (Invitrogen). Cells were harvested 48 h later. The knockdown efficiency of si-KCNQ1OT1 and c-Myc was verified by RT-qPCR.

Cell grouping and plasmid transfection information are shown in Supplementary Table 1.

### Isolation, purification, and identification of EVs

Isolation and purification of EVs: According to the user’s manual, EVs in supernatant of SNU-1 cell medium were obtained through the EVs extraction kit (ExoQuick, SBI, CA, USA). SNU-1 cells were cultured in a 10 cm dish. When cell confluence reached 90%, the medium was replaced with 1640 medium without FBS. Subsequent to 24-h incubation, supernatant of cell medium was collected from 20 mL medium (1 × 10^7^ cells) and centrifuged at 3000 × *g* for 15 min. Then 1000 μL ExoQuick Exosome precipitation solution was supplemented to 1000 μL supernatant. After that, the mixture was frozen at 4 °C for 30 min. Subsequent to 30-min centrifugation at 1500 × *g*, 100 μL sterile 1 × PBS was used to resuspend the EVs precipitate. The supernatant was used as the control without exosome for further experiments.

Identification of EVs: The EV precipitates were identified by a transmission electron microscopy (TEM). Western blot analysis was adopted to identify the protein markers CD9, CD81, CD63, and TSG101 of EVs.

### TEM observation

EVs isolated and purified from tumor were taken out and dropped onto the sealing membrane. The side of Formvar membrane from the copper mesh was placed on the suspension. 2–3 copper meshes were prepared for each EVs sample. After the cover was closed, the copper mesh was allowed to absorb EVs in a dry environment for 20 min. Then 100 μL PBS was added to the sealing membrane. The copper mesh (Formvar film face down) was placed on the PBS with tweezers for 5 min and washed twice. For all steps, the surface of Formvar film remained wet with the other side being dry. The copper mesh was positioned on 50 μL glutaraldehyde (1%) for 5 min before 2-min placing with 100 μL double distilled water (washed 8 times). The copper mesh was plated on 50 μL uranyl oxalate droplet for 5 min and then on 50 μL methylcellulose UA solution for 10 min on ice. After the copper mesh was removed by the stainless steel ring, the excess liquid was gently sucked off with a filter paper, leaving a thin layer of methyl cellulose membrane which was dried in the air for 10 min. The image was observed at 100KV by H7650 TEM (Hitachi, Japan).

### Particle size analysis of Nanosight EVs

Particle size analysis of nanosight nanoparticles: 20 μg EVs were dissolved in 1 mL PBS and vortex for 1 min to keep the EVs evenly distributed. Then the particle size distribution (PSD) of EVs was measured by NanoSight nanoparticle tracking analyzer (NTA, Malvern Instruments, Malvern, UK).

Nanosight (Merkel Technologies Ltd., Israel, NTA version NTA 3.2 Dev Build 3.2.16) NTA was employed to characterize EV concentration. The size distribution and concentration of EVs were determined by ZetasizerNano ZS90 (Malvern instruments, Zetasizer 7.12). The PSD map was established with the *x*-axis representing PSD (nm) and the *y*-axis representing the relative percentage. In addition, PSD map based an intensity was generated according to the particle size (nm) of the *x*-axis and the intensity (a.u.) of the *y*-axis.

### Tracing experiment of EVs in GC cells

EVs were labeled with membrane marking dye PKH67 green fluorescence (Sigma, St Louis, MO, USA). EVs secreted by SNU-1 cells were labeled with PKH67 dye. The labeled EVs were co-cultured with NCI-N87 cells for 30 min, 2 h, and 24 h, respectively. Then NCI-N87 cells were immobilized with 4% paraformaldehyde. Subsequent to 10-min nuclei staining with 4′,6-diamidino-2-phenylindole (DAPI, C1025, 10 μg/mL, Beyotime, Nantong, China), uptake of labeled EVs by recipient GC cells was observed by Nikon eclipse fluorescence microscope (Nikon, Tokyo, Japan).

### Western blot analysis

EV suspension was concentrated or digested with trypsin. The cultured cells were lysed with enhanced Radio-Immunoprecipitation assay cell lysis buffer (BOSTER, Wuhan, Hubei, China) encompassing protease inhibitor at 4 °C for 15 min before 15-min centrifugation at 6000 × *g*. The protein content in the supernatant was determined through a bicinchoninic acid kit (23227, Thermo Fisher Scientific, Waltham, Massachusetts, USA). Sodium dodecyl sulfate polyacrylamide gel electrophoresis gel was prepared before protein denaturation and electrophoresis. Proteins were separated and electroblotted to a polyvinylidene fluoride membrane that underwent 1-h sealing at ambient temperature with 5% bovine serum albumin (BSA) to block the nonspecific binding, and probed overnight with diluted primary antibodies (Abcam, Cambridge, UK) to CD9 (ab195422, 1:1000), CD81 (ab109201, 1:5000), CD63 (ab134045, 1:1000), Tsg101 (ab83, 1:1000), Calnexin (ab22595, 1:1000), c-Myc (ab32072, 1:1000), CLIC1 (ab219265, 1:1000), pho (Y607)-PI3K (1:1000, ab182651), Total-PI3K (1:1000, ab32089), pho (T308)-AKT (1:1000, ab38449), and Total-AKT (1:1000, ab8805) with glyceraldehyde-3-phosphate dehydrogenase (GAPDH) as a normalizer at 4 °C. Afterwards, the membrane was reprobed for 1 h with horseradish peroxidase-tagged goat anti-mouse (ab6808, 1: 2000) and goat anti-rabbit (ab6721, 1: 5000) secondary antibodies (Abcam) at ambient temperature. The membrane was put into electrogenerated chemiluminescence (ECL) reaction solution at ambient temperature before 1-min incubation. Then the excess ECL solution was discarded, followed by membrane sealing with plastic film. The X-ray film was placed in the cassette, followed by 5–10 min-exposure. Subsequent to developing and immobilizing, the image J analysis software was applied for quantification of gray level of each band.

### RT-qPCR

Total RNA extraction was conducted in GC cells or EVs by TRIzol^®^ reagent (15596-018, Solarbio, Beijing, China) before cDNA synthesis. To detect lncRNA and mRNA expression, a PrimeScript™ RT-qPCR Kit (RR047A, TaKaRa, Tokyo, Japan) was applied for reverse transcription. For miRNA detection, Poly(A) Tailing Kit (B532451, Shanghai Sangon Biotechnology Co., Ltd., Shanghai, China; containing universal PCR primer R) was used for reverse transcription to obtain the cDNA of miRNA with PolyA tail. RT-qPCR was conducted on LightCycler 480 system (Roche Diagnostics, Pleasanton, CA, USA) using SYBR^®^ Premix Ex Taq^TM^ II Kit (DRR081, Takara). Primers for amplification were provided by Shanghai General Biotechnology Co., Ltd. (Shanghai, China; Supplementary Table 2). The 2^−△△CT^ method was employed for calculation of the relative transcription levels of target genes that were normalized to GAPDH (for lncRNA and mRNA) and U6 (for miRNA). All investigations involved at least 3 wells, each repeated in triplicate.

### In situ hybridization (ISH)

As per the directions of an ISH kit (BOSTER), paraffin-embedded tissue samples were utilized for ISH to assess KCNQ1OT1 expression. The paraffin slices were dewaxed and hydrated before 10-min immersion in xylene. The slices were soaked in xylene for another 10 min after changing xylene. The slices were positioned in 100%, 75%, and 50% alcohol for 5 min, and then in distilled water for 3 min. Following immobilizing, pre-hybridization, and hybridization, the slices were supplemented with biotinylated rat anti-digoxin and biotinylated peroxidase, and stained with a diaminobenzidine Kit (Solarbio) in the light of the instructions.

### Dual-luciferase reporter assay

The fragment sequence encompassing the action site was obtained. The predicted binding site of KCNQ1OT1 was inserted into pMIR-REPORT luciferase vector (Promega, Madison, WI, USA) for the generation of the luciferase reporter vector wild type (WT)-KCNQ1OT1. Based on this vector, the binding site of KCNQ1OT1 was mutated through a Quick-change site-directed mutagenesis Kit (Agilent Technologies, Palo Alto, CA, USA) to establish the mutant vector mutant (Mut)-KCNQ1OT1.

The fragment sequence encompassing the action site was harvested. CLIC1 3′-untranslated region (UTR) sequence encompassing predicted miR-556-3p binding site was inserted into pMIR-REPORT luciferase vector (Promega) to generate luciferase reporter vector CLIC1-WT. Based on this vector, the binding site of CLIC1 was mutated by Quick-change site-directed mutagenesis Kit (Agilent Technologies) to construct the mutant vector CLIC1-Mut.

HEK-293 cells were cultured in a 24-well plate for 24 h. After reached 50–60% confluence, the cells were co-transfected with Lipofectamine 2000 transfection reagent according to the following combinations: miR-NC/miR-556-3p mimic and CLIC1-WT; miR-NC/miR-556-3p mimic and CLIC1-Mut, miR-NC/miR-556-3p mimic and WT-KCNQ1OT1; miR-NC/miR-556-3p mimic and Mut-KCNQ1OT1. Following 48-h transfection, cell lysate was attained to detect luciferase activity. Relative luciferase activity was evaluated as per the manuals of a dual-luciferase reporter kit (E1910; Promega). Next, the 80–100 μL lysis buffer was added. Subsequent to 30-min passive lysing on ice and a shaker, cells were centrifuged at 4 °C and 12,000 × *g* for 10 min. Then 10 μL supernatant was drawn and positioned on a special 96-well plate for luciferase. The cells were added with 50 μL firefly luciferase buffer (FB) or 50 μL Renilla luciferase buffer (RB) in turn, and then detected by enzyme labeling instrument.

### Immunofluorescence staining

Flag-labeled c-Myc was transfected into GC cells. Following 48-h transfection, EVs were isolated from supernatant of the culture medium and co-cultured with GC cells. After co-culture for 30 min, 2 h, and 24 h severally, cells were immobilized with pre-cooled 4% paraformaldehyde for 20 min. The cells were incubated with Triton X-100 (Sigma) and the membrane was broken for 15 min to increase their permeability. The cells were sealed for 1 h with 5% BSA and probed overnight with anti-FLAG (ab18230, Abcam) primary antibody on the shaking table. The primary antibody was discarded, followed by 1-h cell incubation with conjugated fluorescent secondary antibody at ambient temperature. After the secondary antibody was discarded, cells were stained with DAPI (10 μg/mL, C1002, Beyotime) without light for 15 min. Then anti-fluorescence attenuation sealing agent was dripped onto the glass slide. After the cover glass was covered, the diagonal of the film was sealed with nail polish, and the sample was observed by fluorescence microscope (Leica Biosystems, Shanghai, China).

### Cell counting kit (CCK)-8

Cells were incubated in a 96-well plate (1 × 10^3^ cells/well) with 100 μL medium encompassing 10% FBS for 3 days. Then, cell number was evaluated as per the protocols of a CCK-8 kit (K1018, Apexbio, USA). Next, 10 μL CCK-8 solution was supplemented to each well for another 4-h culture at 37 °C. Optical density was assessed at 450 nm on days 1, 2, and 3. Five parallel wells were set up for each experiment. Proliferation rates relative to controls were applied to plot cell growth curves that were examined using one-way analysis of variance (ANOVA).

### Flow cytometry

The apoptosis rate of GC cells was detected using Annexin V-allophycocyanin (APC) apoptosis detection kit (556547, BD Pharmingen, San Jose, USA). GC cells were made into a suspension of 1 × 10^6^ cells/mL with 1× Binding Buffer. Falcon tube was supplemented with 100 μL cell suspension and 5 μL Annexin V in the light of the kit instructions, mixed gently, and kept in dark at ambient temperature (20–25 °C) for 15 min. Subsequent to once cell washing with 1× Binding Buffer, the supernatant was removed. Cell precipitate was resuspended with 100 μL of 1× Binding Buffer, and supplemented with 5 μL propidium iodide, followed by 15-min placing in dark at ambient temperature (20–25 °C). 400 μL of 1× Binding Buffer was supplemented to each test tube, and the results were determined using a FACScan flow cytometry system (Becton Dickinson, San Diego, CA, USA) within 1 h.

### Scratch test

On the bottom of the 6-well plate, a horizontal line was drawn through a ruler and a marker every 0.5–1 cm with that each well shall pass through at least five lines. GC cells (1 × 10^6^) were added to the 6-well plate and grew to fusion. Cells were incubated overnight in medium with or without EVs. Then, a sterile gun head (200 μL) was applied to scratch horizontal lines perpendicular to the back. Cells were removed and medium without serum was supplemented before cell incubation in a 5% CO_2_ and 37 °C incubator. After 0, 12, and 24 h of culture, the growth and metastasis of GC were observed and recorded by inverted microscope. The migration closure was calculated: Migration area (%) = (A0 − An)/A0 × 100, where A0 represented the initial wound area and An indicated the wound area at the time of measurement.

### Transwell migration and invasion experiment

In order to analyze cell migrative ability, 2 × 10^5^ transfected cells were suspended in DMEM (200 μL) without serum and supplemented to Transwell apical chamber without Matrigel reagent (356234, Becton, Dickinson and Company, NJ, USA).

To analyze cell invasive ability, Matrigel reagent from Becton, Dickinson and Company was diluted in DMEM without serum (1:10). The diluted Matrigel (100 μL) was supplemented to Transwell apical chamber before more than 30 min of culture. Then the cells were seeded into the upper chamber coated with Matrigel (BD Pharmingen). For both assays, DMEM encompassing 10% FBS (600 μL) was supplemented to the basolateral chamber before 24-h incubation at 37 °C. Subsequent to 15-min cell immobilizing and 15-min 0.1% crystal violet staining, the positive cells were observed by inverted light microscope (Carl Zeiss, Jena; Evotec Biosystems, Hamburg, Germany) and photographed. The positive cells were counted by ImageJ software.

### Statistical analysis

SPSS 21.0 (IBM Corp. Armonk, NY, USA) was employed in statistical analysis. The Measurement data are summarized as mean ± standard deviation. Paired *t*-test was used to analyze the data between cancer tissues and adjacent normal tissues. Unpaired *t*-test was conducted for comparison between the other two groups. One-way ANOVA was utilized to compare among multiple groups, while two-way ANOVA was applied for comparing the data at different time points, followed by Tukey’s post-hoc test. *p* < 0.05 was considered to be statistically significant difference.

## Results

### The transcription factor c-Myc and its downstream lncRNA KCNQ1OT1 were highly expressed in GC tissues

**Fig. 1:**
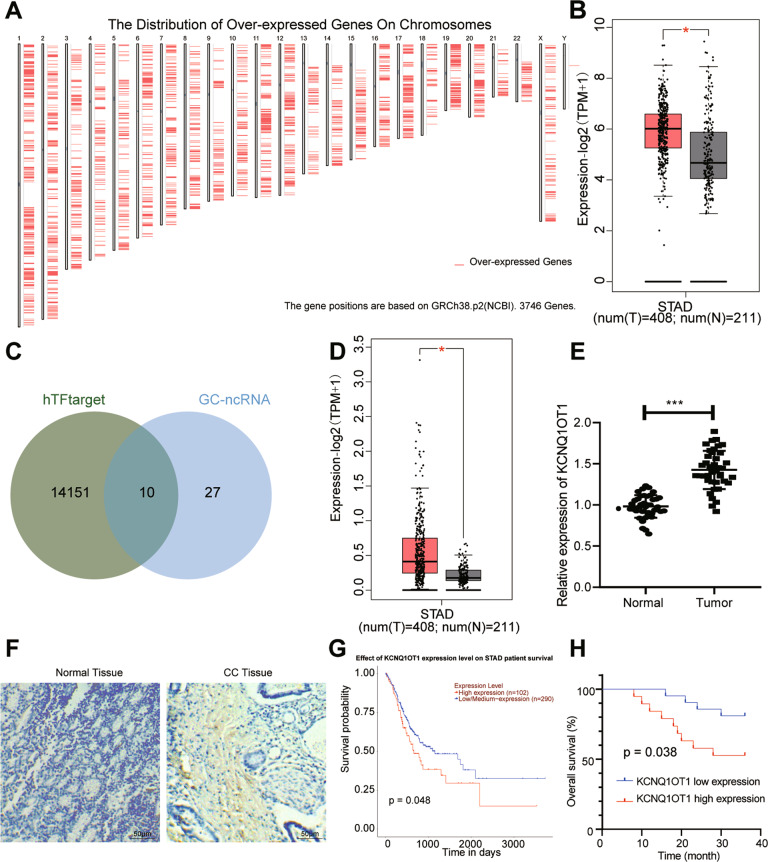
c-Myc and KCNQ1OT1 expression is high in GC tissues. **A** GEPIA (http://gepia2.cancer-pku.cn/) was used to analyze the chromosomal location of upregulated 3746 genes in GC. **B** GEPIA website was used to analyze the expression of c-Myc in adjacent normal tissues and GC tissues (Red represents GC, *N* = 408; Gray represents adjacent normal tissues, *N* = 211). **C** Venn diagram of GC-related ncRNAs in GeneCards database (https://www.genecards.org/) and hTFtargets (http://bioinfo.life.hust.edu.cn/hTFtarget#!/)-predicted candidate targets of c-Myc formed by jvenn (http://jvenn.toulouse.inra.fr/app/example.html). **D** GEPIA website was used to assess the expression of KCNQ1OT1 in adjacent normal tissues and GC tissues (Red represented GC, *N* = 408; Gray represented adjacent normal tissues, *N* = 211). E, Expression of KCNQ1OT1 in adjacent normal tissues and GC tissues determined by RT-qPCR (*N* = 40). **F** ISH was conducted to detect the expression of KCNQ1OT1 in adjacent normal tissues and GC tissues (scale bar: 25 μm). **G** The relationship between the expression of KCNQ1OT1 and the prognosis of GC patients analyzed through the website of Uaclan. **H** The Kaplan–Meier method was used to analyze the relationship between the expression of KCNQ1OT1 and the overall survival of GC patients. *p < 0.05 compared with the two groups.

To acknowledge the mechanism in GC, bioinformatics analysis was conducted. Totally 3746 genes were documented to be overexpressed in the TCGA database using the GEPIA database (Fig. 1A). Moreover, we discovered that c-Myc was highly expressed in GC by analyzing the TCGA database with GEPIA (Fig. 1B). To explore the mechanism of GC growth and metastasis, we predicted 14151 targeting factors of transcription factor c-Myc through the bioinformatics prediction website hTFtargets, and obtained 37 GC-related ncRNAs through the GeneCards database. Ten candidate factors (MIR200B, MIR200C, MIR25, TERC, MIR106B, MIR93, MIR148A, KCNQ1OT1, MIR141, and MIR150) were obtained by the intersection of GC-related ncRNA and candidate targeting factors in hTFtargets through jvenn tool (Fig. 1C), among which TERC and KCNQ1OT1 were lncRNAs. It was displayed that KCNQ1OT1 was highly expressed in GC by analyzing the TCGA database with GEPIA (Fig. 1D). RT-qPCR results showed higher KCNQ1OT1 expression in GC tissues than the adjacent normal tissues (Fig. 1E). ISH data showed upregulation of KCNQ1OT1 in GC tissues (Fig. 1F). GEPIA website analysis demonstrated that the higher KCNQ1OT1 expression, the lower the survival rate of GC patients (Fig. 1G). The Kaplan–Meier method was used to analyze the relationship between the expression of KCNQ1OT1 and the overall survival of GC patients. The specimens were divided into a high expression group and a low expression group according to the mean value of KCNQ1OT1 expression in GC. The results suggested that patients with high expression of KCNQ1OT1 had a shorter overall survival than patients with low expression of KCNQ1OT1 (*p* < 0.05; Fig. 1H). Next, JASPAR was adopted to predict binding sites of c-Myc to KCNQ1OT1 (Supplementary Fig. 1). Collectively, both transcription factor c-Myc and its downstream lncRNA KCNQ1OT1 were upregulated in GC tissues, which might interact with each other to modulate the growth and metastasis of GC.

### Tumor-derived EVs could be internalized by GC cells, thereby enhancing the metastatic ability of GC cells

**Fig. 2:**
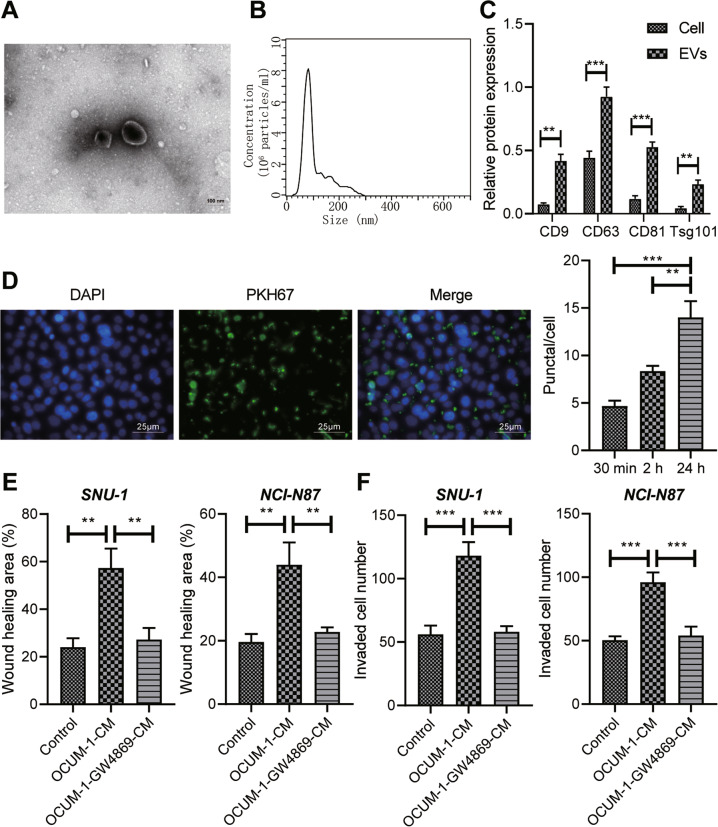
Tumor-derived EVs induce invasion and migration of GC cells. **A** The morphology of EVs observed by a TEM (×10000). **B** Analysis of particle size of EVs by NanoSight nanoparticle tracking analyzer. **C** The expression of CD9, CD63, CD81, Calnexin, and TSG101 in GC cells and EVs detected by Western blot analysis. **D** Observation of internalized EVs in GC cells by immunofluorescence staining. **E** The migration of GC cells detected by scratch test. **F** The invasion of GC cells detected by Transwell assay. * represents the comparison between the two groups. *p < 0.05; **p < 0.01; ***p < 0.001.

We first examined the effects of EVs derived from GC cell line SNU-1 on the growth and metastasis of GC to confirm our conjecture. From the results of TEM, EVs were round or cup-shaped (Fig. 2A). Nanosight analysis indicated that the size of tumor-derived EVs was between 30 nm and 300 nm (Fig. 2B). Moreover, CD9, CD63, CD81, and TSG101 expression in GC cell line SNU-1 was low or not, but were high in EVs, whereas the endoplasmic reticulum membrane protein Calnexin was highly expressed in cells, but lowly or not expressed in EVs (Fig. 2C; Supplementary Fig. 2A). Then, GC cell line SNU-1 was incubated with EVs. EVs were labeled with PKH67 (green fluorescence), and GC cell nuclei were labeled with DAPI (blue fluorescence). Fluorescence microscopy displayed that the EVs surrounded the nucleus after entering the cells (Fig. 2D).

In order to explore the influence of EVs on migrative and invasive capabilities of SNU-1 cells, we added or not added EVS secretion inhibitor GW4869 into SNU-1 cells. SNU-1 and NCI-N87 cells were incubated with the supernatant of conditioned medium (CM) of each group. Scratch test and Transwell test exhibited that migrative and invasive ability of SNU-1 and NCI-N87 cells incubated with SNU-1-CM were significantly enhanced, while was opposite after incubation with SNU-1-GW4869-CM (Fig. 2E, F). Conclusively, GC cells could internalize EVs, which enhanced the metastatic ability of GC cells.

### EVs-c-Myc could promote the proliferative, migrating, and invasive potential of GC cells

**Fig. 3:**
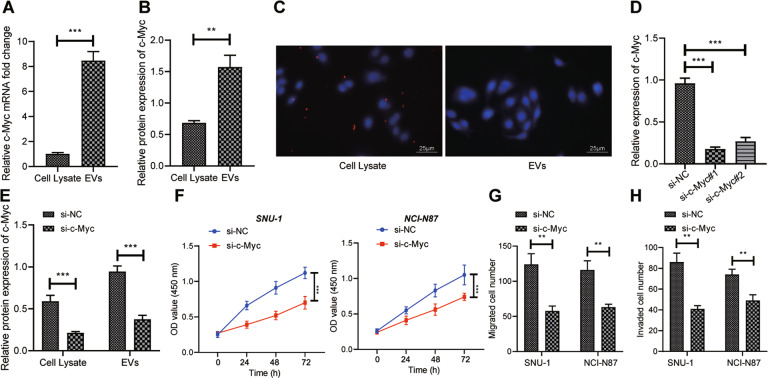
EVs-c-Myc accelerates proliferation, migration, and invasion of GC cells. **A** RT-qPCR was used to detect the mRNA expression of c-Myc in GC cells and EVs. **B** The protein expression of c-Myc in GC cells and EVs determined by Western blot analysis. **C** The expression of Flag-c-Myc in GC cells detected by immunofluorescence (Flag labeling was red fluorescence, DAPI labeling was blue fluorescence; scale bar: 50 μm). **D** Detection of knockdown efficiency of two si-c-Myc sequences by RT-qPCR. **E** Western blot analysis was used to detect the protein expression of c-Myc in SNU-1 cells and EVs after knockdown of c-Myc. **F** CCK-8 was used to check the proliferation of SNU-1 and NCI-N87 cells after knockdown of c-Myc in EVs. **G** Transwell assay was used to test the migration of SNU-1 and NCI-N87 cells after incubation with EVs from GC cells with knockdown of c-Myc. **H** The invasion of SNU-1 and NCI-N87 cells after incubation with EVs from GC cells with knockdown of c-Myc detected by Transwell assay. * represents the comparison between the two groups. *p < 0.05; **p < 0.01. The cell experiment was repeated 3 times.

Our previous results manifested that GC cells could internalize EVs. Then, we explored whether c-Myc came from EVs and promoted the growth and metastasis of GC cells. As for RT-qPCR and Western blot analysis, c-Myc upregulation was observed in EVs in contrast to GC cells (Fig. 3A, B; Supplementary Fig. 2B).

To further confirm that EVs delivered c-Myc to GC cells, GC cells were transfected Flag-labeled c-Myc. EVs were isolated from the supernatant and incubated with GC cells for 24 h. On the basis of immunofluorescence results, red fluorescence was found in untransfected GC cells (Fig. 3C), indicating that c-Myc could be transferred from EVs to GC cells.

Furthermore, c-Myc was knocked down in SNU-1 GC cells. RT-qPCR was conducted to verify the knockdown efficiency of two si-c-Myc sequences in SNU-1 cells. The results demonstrated that both of them effectively reduced c-Myc expression (Fig. 3D). After that, the siRNA sequence with the highest knockdown efficiency was applied to knock down c-Myc in NCI-N87 and SNU-1 cells. According to the Western blot analysis results, c-Myc expression was lowered in cells and EVs by si-c-Myc (Fig. 3E; Supplementary Fig. 2C). Then, NCI-N87 and SNU-1 cells were incubated with EVs. As reflected by CCK-8 and Transwell assays, EVs derived from c-Myc-knockdown cells inhibited NCI-N87 and SNU-1 cell viability (Fig. 3F), migration, and invasion (Fig. 3G, H). Altogether, tumor-derived EVs could transfer c-Myc to GC cells, thereby enhancing GC cell viability, invasion, and migration.

### c-Myc elevated KCNQ1OT1 expression to facilitate GC cell viability, migration, and invasion

**Fig. 4:**
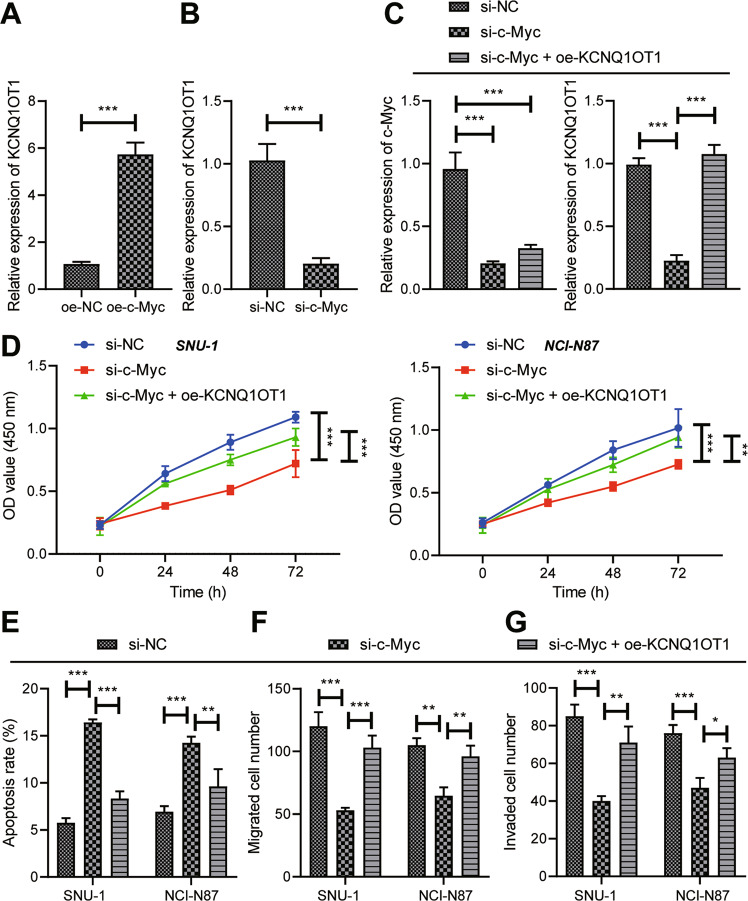
c-Myc silencing inactivates KCNQ1OT1 to dampen GC cell viability, migration, and invasion. **A** The expression of KCNQ1OT1 in GC cells overexpressing c-Myc determined by RT-qPCR. **B** RT-qPCR was used to detect the expression of KCNQ1OT1 in GC cells knocking down c-Myc. GC cells were transfected with si-NC, si-c-Myc, or si-c-Myc + oe-KCNQ1OT1. **C** RT-qPCR was used to detect the expression of c-Myc and KCNQ1OT1 in cells. **D** Cell viability determined by CCK-8. **E** Cell apoptosis rate measured by flow cytometry. **F** Cell migration detected by Transwell assay. **G** The invasion of GC cells assessed by Transwell assay. * represents the comparison between the two groups. *p < 0.05; **p < 0.01; ***p < 0.001. The cell experiment was repeated 3 times.

c-Myc was overexpressed or knocked down in SNU-1 GC cells to investigate whether c-Myc mediated KCNQ1OT1 expression in GC cells. RT-qPCR results documented that overexpression of c-Myc enhanced KCNQ1OT1 expression in GC cells (Fig. 4A), which was opposite after knockdown of c-Myc (Fig. 4B), indicating that c-Myc could augment KCNQ1OT1 expression.

In order to verify whether c-Myc could facilitate the growth and metastasis of GC by activating KCNQ1OT1, KCNQ1OT1 was overexpressed in GC cells after c-Myc knockdown. RT-qPCR results manifested that c-Myc and KCNQ1OT1 expression in cells silencing c-Myc was evidently decreased. In the presence of si-c-Myc, c-Myc expression was not changed, but KCNQ1OT1 expression was substantially increased by further oe-KCNQ1OT1 treatment (Fig. 4C). Furthermore, si-c-Myc diminished cell proliferative (Fig. 4D), migrating (Fig. 4F), and invasive (Fig. 4G) potential, but augmented cell apoptosis (Fig. 4E), which was negated by si-c-Myc + oe-KCNQ1OT1 treatment. In summary, c-Myc could induce GC cell viability, migration, and invasion by upregulating KCNQ1OT1.

### KCNQ1OT1 bound to miR-556-3p to enhance GC cell viability, migrative, and invasive ability

**Fig. 5:**
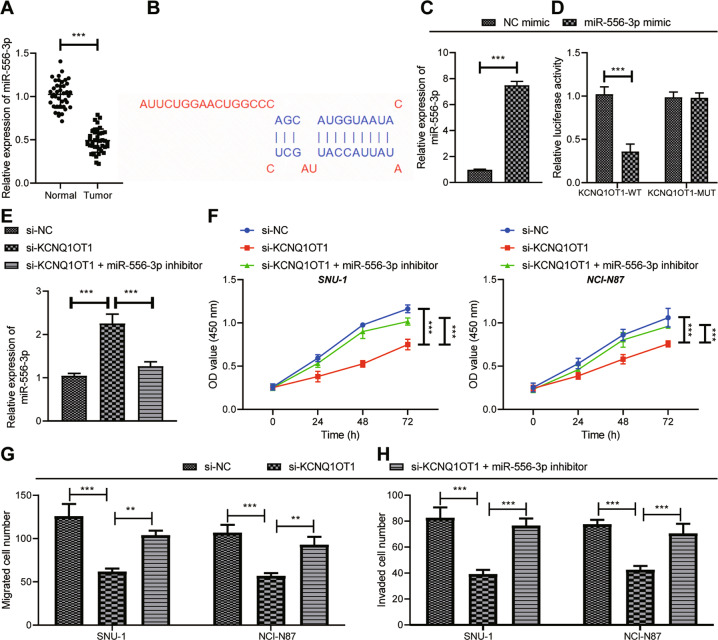
KCNQ1OT1 knockdown induces GC cell viability, invasion, and migration by upregulating miR-556-3p. **A** RT-qPCR to measure the expression of miR-556-3p in adjacent normal tissues and GC tissue (*N* = 40). **B** LncBase database (http://carolina.imis.athena-innovation.gr/diana_tools/web/index.php?r = lncbasev2%2Findex) predicting the binding site between KCNQ1OT1 and miR-556-3p. **C** The expression of miR-556-3p after miR-556-3p mimic treatment detected by RT-qPCR. **D** Dual-luciferase reporter assay was used to detect the binding relationship of KCNQ1OT1 to miR-556-3p. GC cells were transfected with si-NC, si-KCNQ1OT1, or si-KCNQ1OT1 + miR-556-3p inhibitor. **E** The expression of miR-556-3p detected by RT-qPCR. **F** CCK-8 was used to measure cell viability. **G** Cell migration determined by Transwell assay. **H** The invasion of GC cells detected by Transwell assay. * represents the comparison between the two groups. *p < 0.05; **p < 0.01; ***p < 0.001. All experiments were repeated 3 times.

To further explore the downstream targeted miRNAs of KCNQ1OT1, 1845 miRNAs binding to lncRNA were predicted by LncBase database, and 444 and 213 GC-related miRNAs were predicted by MnDR v3.1 and MISIM v2.0, respectively. Totally 27 candidate miRNAs were obtained from the intersection of miRNAs binding to lncRNA and GC-related miRNAs (Supplementary Fig. 3A). The expression of these 27 candidate miRNAs in GC samples was searched through dbDEMC 2.0 website, the results of which showed that miR-664b-3p, miR-204-5p, miR-218-5p, miR-23c, and miR-556-3p were markedly lowly expressed in GC (Supplementary Fig. 3B). Moreover, miR-556-3p expression was strikingly low in breast cancer and bladder cancer through further predicting miR-556-3p expression in other tumors (Supplementary Fig. 3C). As for RT-qPCR results, in contrast to adjacent normal tissues, miR-556-3p expression in GC tissues was lower (Fig. 5A).

Therefore, in order to further explore whether KCNQ1OT1 bound to miR-556-3p and affected the growth and metastasis of GC cells, we predicted the binding sites of KCNQ1OT1 with miR-556-3p by LncBase database (Fig. 5B), and constructed a GC cell model overexpressing miR-556-3p (Fig. 5C). As indicated by dual-luciferase reporter assay results, the luciferase activity of the wt-KCNQ1OT1 was appreciably diminished by miR-556-3p mimic (Fig. 5D). In order to investigate the impact of KCNQ1OT1/miR-556-3p axis on GC cells, KCNQ1OT1 and miR-556-3p was simultaneously knocked down in GC cells. RT-qPCR results depicted that elevated miR-556-3p level was noticed in cells silencing KCNQ1OT1, which was opposite following si-KCNQ1OT1 + miR-556-3p inhibitor treatment (Fig. 5E). As illustrated in Fig. 5F–H, cell viability, migrative, and invasive capabilities were reduced after silencing KCNQ1OT1, which was counteracted by further miR-556-3p inhibitor treatment. In a short, knockdown of KCNQ1OT1 could upregulate miR-556-3p to repress GC cell viability, migration, and invasion.

### KCNQ1OT1 bound to miR-556-3p to upregulate CLIC1 in GC cells

**Fig. 6:**
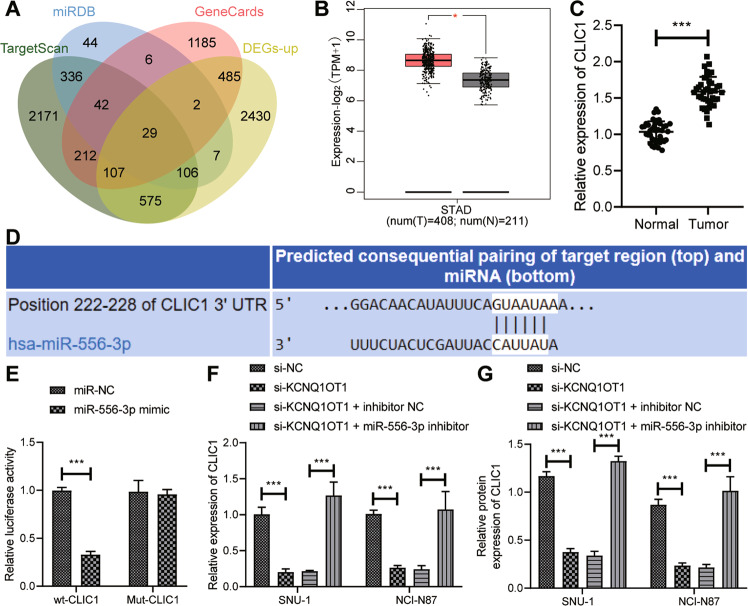
KCNQ1OT1 binds to miR-556-3p to augment CLIC1 expression in GC cells. **A** Venn diagram of target genes of miR-556-3p in TargetScan (http://www.targetscan.org/vert_72/) and miRDB (http://www.mirdb.org/), GC-related genes in GeneCards, and upregulated genes. **B** Prediction of CLIC1 expression in GC and adjacent normal tissues by GEPIA website (Red represented GC, *N* = 408; Gray represented adjacent normal tissues, *N* = 211). C, Detection of CLIC1 expression in GC and adjacent normal tissues by RT-qPCR (*N* = 40). **D** Targetscan website predicting the binding sites of miR-556-3p and CLIC1. **E** Dual-luciferase reporter assay was used to verify the targeting relationship between miR-556-3p and CLIC1. **F** The mRNA expression of CLIC1 detected by RT-qPCR after si-KCNQ1OT1 and miR-556-3p inhibitor treatment. **G** Western blot was used to detect the protein expression of CLIC1 after si-KCNQ1OT1 and miR-556-3p inhibitor treatment. * represents the comparison between the two groups. *p < 0.05; **p < 0.01; ***p < 0.001. All experiments were repeated 3 times.

In order to further explore the downstream regulatory factors of miR-556-3p involved in GC cell migration, we predicted the target genes of miR-556-3p by TargetScan and miRDB. Then, jvenn tool was employed to find the overlapping parts of target genes, GC-related genes, and upregulated genes to obtained 29 candidate genes (Fig. 6A). GEPIA analysis of TCGA database exhibited that CLIC1 was highly expressed in GC (Fig. 6B). RT-qPCR demonstrated that versus adjacent normal tissues, CLIC1 expression was high in GC tissues (Fig. 6C).

To further confirm that CLIC1 was targeted by miR-556-3p, we predicted the binding site of miR-556-3p to CLIC1 by TargetScan website and mutated the binding site (Fig. 6D). As reflected by dual-luciferase reporter assay results, the luciferase activity of wt-CLIC1 was curtailed by miR-556-3p mimic (Fig. 6E). Furthermore, lowered CLIC1 expression was detected after KCNQ1OT1 silencing, which was nullified by further miR-556-3p inhibitor treatment (Fig. 6F, G; Supplementary Fig. 2D). In summary, KCNQ1OT1 promoted CLIC1 expression by binding to miR-556-3p in GC cells.

### miR-556-3p down-regulated CLIC1 and inhibited PI3K/AKT pathway to reduce GC cell viability, migration, and invasion

**Fig. 7:**
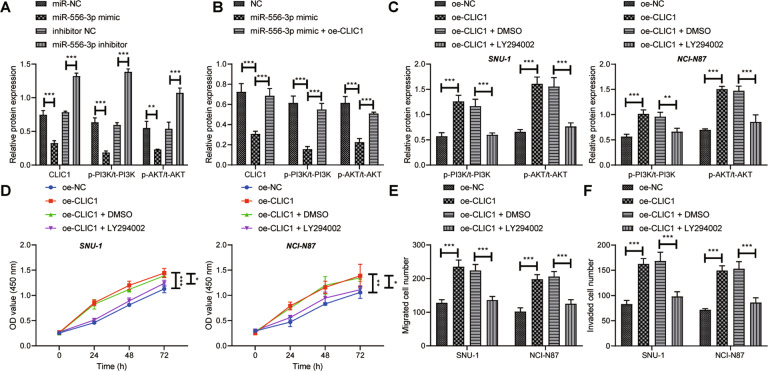
miR-556-3p/CLIC1/PI3K/AKT axis manipulates the viability, invasion, and migration of GC cells. **A** The protein expression of CLIC1, PI3K, and AKT and phosphorylation of PI3K and AKT after overexpression or inhibition of miR-556-3p detected by Western blot analysis. **B** The protein expression of CLIC1, PI3K, and AKT and phosphorylation of PI3K and AKT after overexpression miR-556-3p and CLIC1 determined by Western blot analysis. **C** Western blot analysis was used to detect the effect of AKT inhibitor LY294002 on the protein expression of PI3K and AKT and phosphorylation of PI3K and AKT. **D** CCK-8 was used to detect cell proliferation. **E** Cell migration detected by Transwell assay. **F** The invasion of GC cells measured by Transwell assay. * represents the comparison between two groups. *p < 0.05; **p < 0.01; ***p < 0.001. All experiments were repeated 3 times.

It has been documented that CLIC1 targets the activation of PI3K/AKT pathway [15]. miR-556-3p was overexpressed or knocked down in GC cells to verify whether miR-556-3p blocked PI3K/AKT pathway by downregulating CLIC1. Western blot analysis documented that miR-556-3p mimic caused decline of CLIC1 expression, PI3K phosphorylation, and AKT phosphorylation, while miR-556-3p inhibitor triggered the opposite trends (Fig. 7A).

Western blot analysis depicted reduction of phosphorylation of PI3K and AKT after miR-556-3p mimic treatment, which was counteracted by treatment with miR-556-3p mimic + oe-CLIC1 (Fig. 7B). For exploring the effect of CLIC1/PI3K/AKT axis on GC cells, AKT inhibitor LY294002 was adopted to treat GC cells while overexpressing CLIC1. Based on Western blot analysis results, phosphorylation of PI3K and AKT following CLIC1 overexpression was dramatically increased, which was memorably annulled by further LY294002 treatment (Fig. 7C). Meanwhile, augmented cell viability (Fig. 7D), migrating (Fig. 7E), and invasive (Fig. 7F) abilities were noted in CLIC1-overexpressed GC cells, which was reversed subsequent to further LY294002 treatment. Taken together, miR-556-3p disrupted PI3K/AKT pathway by downregulating CLIC1 to dampen the viability, migration, and invasion of GC cells.

## Discussion

GC is often diagnosed at the advanced stage with a median survival of the affected patients less than 1 year [16]. Additionally, in spite of new advances in understanding the biology of GC, the treatment of patients with advanced GC remains a major problem [17]. Hence, there is a continued need to explore the use of novel treatments for GC. Tumor-derived EVs have been manifested to change the tumor microenvironment, thereby modulating cancer progression that is likely to trigger drug resistance and cancer recurrence [18]. In this context, our work intended to explore the role of tumor-derived EVs in GC development and whether tumor-derived EVs carried c-Myc to impact GC cell processes. As a result, our data illustrated that tumor-derived EVs could augment KCNQ1OT1 expression and reduce miR-556-3p expression by transferring c-Myc to GC cells to upregulate CLIC1 and activate the PI3K/AKT pathway, so as to enhance GC cell proliferating, migrating, and invasive potential.

Initially, our data ascertained the presence of upregulated c-Myc and KCNQ1OT1 in GC cells, and that c-Myc overexpression caused upregulation of KCNQ1OT1 in GC cells. Consistently, Liu et al. observed the upregulation of c-Myc in primary GC tissues [10]. Also, the research conducted by Zhong et al. showed obvious augment of KCNQ1OT1 expression in GC tissues and cells [19], which was concordant with our data. Partially concurrent with our results, c-Myc silencing triggered down-regulation of KCNQ1OT1 by diminishing the ability of c-Myc to bind to the KCNQ1OT1 promoter [11]. Moreover, our further cell experiments elucidated that silencing c-Myc or KCNQ1OT1 contributed to decline of GC cell proliferative, migrating, and invasive capabilities but enhancement of their apoptosis. Similarly, it was noted in a prior research that c-Myc knockdown curtailed GC cell growth and proliferation but induced their apoptosis [20]. Also, Sun et al. found that c-Myc overexpression led to accelerated GC cell proliferation and invasion [21]. Additionally, another work manifested that c-Myc silencing dampened proliferation, migration, and invasiveness of GC cells [22]. Ectopic KCNQ1OT1 expression also subdued GC cell proliferative, migrating, and invasive potentials, but facilitated their apoptosis [12, 19, 23], which was in consistent with our results.

As widely recognized, lncRNA can bind to miRNAs to function as competing endogenous RNAs or sponge for miRNAs that post-transcriptionally manipulated expression of target genes [24]. Similarly, our further mechanical analysis ascertained that KCNQ1OT1 lowered miR-556-3p expression by binding to miR-556-3p to upregulate CLIC1 and to activate the PI3K/AKT pathway, thus facilitating GC cell proliferative, migrating, and invasive abilities but repressing their apoptosis. A prior study elaborated miR-556-3p as an anti-oncogene in lung cancer by reducing cell proliferative, invasive, and migrating capabilities but enhancing cell apoptosis [13]. Besides, CLIC1 expression was high in GC tissues, and silencing CLIC1 could culminate in diminishment of GC cell invasive and migrating potentials [25]. Also, corroborating findings were reported by another work that CLIC1 silencing by targeted-siRNA potently dampened GC cell invasiveness and migration and facilitated their apoptosis in vitro by disrupting the PI3K/AKT pathway [26]. Furthermore, blocking the PI3K/AKT pathway was capable of curtailing GC cell invasiveness and migration [27].

Another central finding in our work was that tumor-derived EVs augmented GC cell proliferative, invasive, and migrating by delivering c-Myc. It is well-known that EVs can be released by various types of cells, including tumor cells [28]. Moreover, EVs assume a pivotal role in intercellular and distal communication to trigger numerous processes that facilitate tumor progression and metastases [29]. Consistently, the research of Zhang et al. identified that tumor-derived EVs could accelerate GC cell migration [30]. Also, GC cell-derived EVs could enhance GC proliferation, invasion, and migration [8]. EVs can be secreted in both physiological and pathological environment and can deliver multiple molecules, such as proteins, metabolites, RNA, miRNAs, and DNA, to distant sites throughout the body [31]. Intriguingly, our results found that tumor-derived EVs could shuttle c-Myc into GC cells. Similarly, a previous work clarified that tumor-derived EVs could accelerate lung bronchial cell proliferation to induce lung cancer progression by carrying c-Myc [9].

**Fig. 8:**
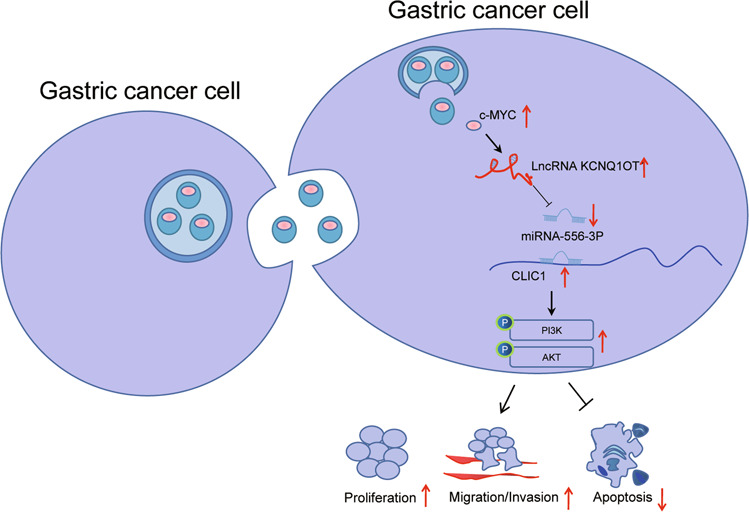
Molecular mechanism of tumor-derived extracellular vesicles delivering c-Myc affects gastric cancer growth and metastasis via the KCNQ1OT1/miR-556-3p/CLIC1 axis. Tumor-derived extracellular vesicles carrying c-Myc blocked miR-556-3p expression by upregulating KCNQ1OT1 to elevate CLIC1 expression,thus activating the PI3K/AKT pathway and accelerating gastric cancer cell proliferation, invasion, and migration.

To conclude, our study suggests the tumor-promoting potential of tumor-derived EVs in GC via c-Myc delivery. Specifically, tumor-derived EVs carrying c-Myc blocked miR-556-3p expression by upregulating KCNQ1OT1 to elevate CLIC1 expression, thus activating the PI3K/AKT pathway and accelerating GC cell proliferation, invasion, and migration (Fig. 8). This study supports the potential application of tumor-derived EVs carrying c-Myc in the therapeutic modality for GC as a diagnostic biomarker and a novel target for pharmacological and genetic manipulation. Further investigation is essential for the validation of the tumor-promoting effects of EVs directly depending on c-Myc pathway due to the complicated components of EVs.

## Supplementary information

Supplementary materials

aj-checklist

## Data Availability

The original contributions presented in the study are included in the article/supplementary material, further inquiries can be directed to the corresponding authors.
