# Supplementary material for: Tumor-derived extracellular vesicles shuttle c-Myc to promote gastric cancer growth and metastasis via the KCNQ1OT1/miR-556-3p/CLIC1 axis
Source: Cell Death Dis. 2022 Mar 8;13(3):217. doi: 10.1038/s41419-021-04446-5 (PMC8904444; doi:10.1038/s41419-021-04446-5)
Supplement: Supplementary file 1 — Supplementary materials [file 41419_2021_4446_MOESM1_ESM.pdf]

Matrix ID	Name	Score	Relative sc	Sequence ID	Start	End	Strand	Predicted sequence
MA0147.3	MYC	5.56161	0.783958	KCNQ1OT1	1481	1492	+	GATCACCTGCCA
MA0147.3	MYC	5.37616	0.780086	KCNQ1OT1	742	753	+	TCCCATCTGCAC
MA0147.3	MYC	5.02114	0.772674	KCNQ1OT1	2271	2282	-	TCCCACTTGTTT
MA0147.3	MYC	4.90559	0.770262	KCNQ1OT1	1488	1499	+	TGCCAGGTGAGA
MA0147.3	MYC	4.85699	0.769247	KCNQ1OT1	303	314	-	CCCCGGGTGCCC
MA0147.3	MYC	4.67598	0.765468	KCNQ1OT1	1981	1992	-	AAACAGGTGCAG
MA0147.3	MYC	4.40172	0.759743	KCNQ1OT1	1151	1162	-	CACCATGCGGAC
MA0147.3	MYC	4.02794	0.751939	KCNQ1OT1	793	804	+	GGCCGCGTCCCA
MA0147.3	MYC	3.99718	0.751297	KCNQ1OT1	442	453	+	CTCCGCGCGGTC
MA0147.3	MYC	3.96725	0.750672	KCNQ1OT1	1151	1162	+	GTCCGCATGGTG
MA0147.3	MYC	3.91904	0.749666	KCNQ1OT1	2361	2372	+	AACCATATGGTT
MA0147.3	MYC	3.91904	0.749666	KCNQ1OT1	2361	2372	-	AACCATATGGTT
MA0147.3	MYC	3.83469	0.747905	KCNQ1OT1	1488	1499	-	TCTCACCTGGCA
MA0147.3	MYC	3.76414	0.746432	KCNQ1OT1	1078	1089	+	GTCCACATCCTG
MA0147.3	MYC	3.74637	0.746061	KCNQ1OT1	13	24	+	CGCCGCGTCGCC
MA0147.3	MYC	3.7437	0.746005	KCNQ1OT1	1981	1992	+	CTGCACCTGTTT
MA0147.3	MYC	3.64551	0.743955	KCNQ1OT1	760	771	-	GGCCACGCTGTC
MA0147.3	MYC	3.63475	0.74373	KCNQ1OT1	1481	1492	-	TGGCAGGTGATC
MA0147.3	MYC	3.54062	0.741765	KCNQ1OT1	442	453	-	GACCGCGCGGAG
MA0147.3	MYC	3.2346	0.735376	KCNQ1OT1	682	693	+	TGCCCCGCGGCC
MA0147.3	MYC	2.99756	0.730428	KCNQ1OT1	760	771	+	GACAGCGTGGCC
MA0147.3	MYC	2.99397	0.730353	KCNQ1OT1	682	693	-	GGCCGCGGGGCA
MA0147.3	MYC	2.94963	0.729427	KCNQ1OT1	13	24	-	GGCGACGCGGCG
MA0147.3	MYC	2.58065	0.721724	KCNQ1OT1	1882	1893	+	AGCCAAGAGCCT
MA0147.3	MYC	2.47427	0.719503	KCNQ1OT1	138	149	+	CTCCCGGTGCGC
MA0147.3	MYC	2.45637	0.719129	KCNQ1OT1	2271	2282	+	AAACAAGTGGGA
MA0147.3	MYC	2.43312	0.718644	KCNQ1OT1	921	932	+	ATCCATGTTGTG
MA0147.3	MYC	2.43167	0.718613	KCNQ1OT1	623	634	-	GCCCAGGTCCGT
MA0147.3	MYC	2.39791	0.717909	KCNQ1OT1	101	112	+	CTCAGCGTGGTC
MA0147.3	MYC	2.10998	0.711897	KCNQ1OT1	877	888	+	CGCATCGTGGTT
MA0147.3	MYC	2.09285	0.71154	KCNQ1OT1	1009	1020	-	TCCCACCTCCGA
MA0147.3	MYC	1.91343	0.707794	KCNQ1OT1	101	112	-	GACCACGCTGAG
MA0147.3	MYC	1.69474	0.703229	KCNQ1OT1	827	838	-	CACCATGAGAAC
MA0147.3	MYC	1.66784	0.702667	KCNQ1OT1	218	229	+	CTCCTCGGGGCT

Supplementary Fig. 1 Prediction of the binding sites between c-Myc and KCNQ1OT1 by JASPAR (http://jaspar.genereg.net/).

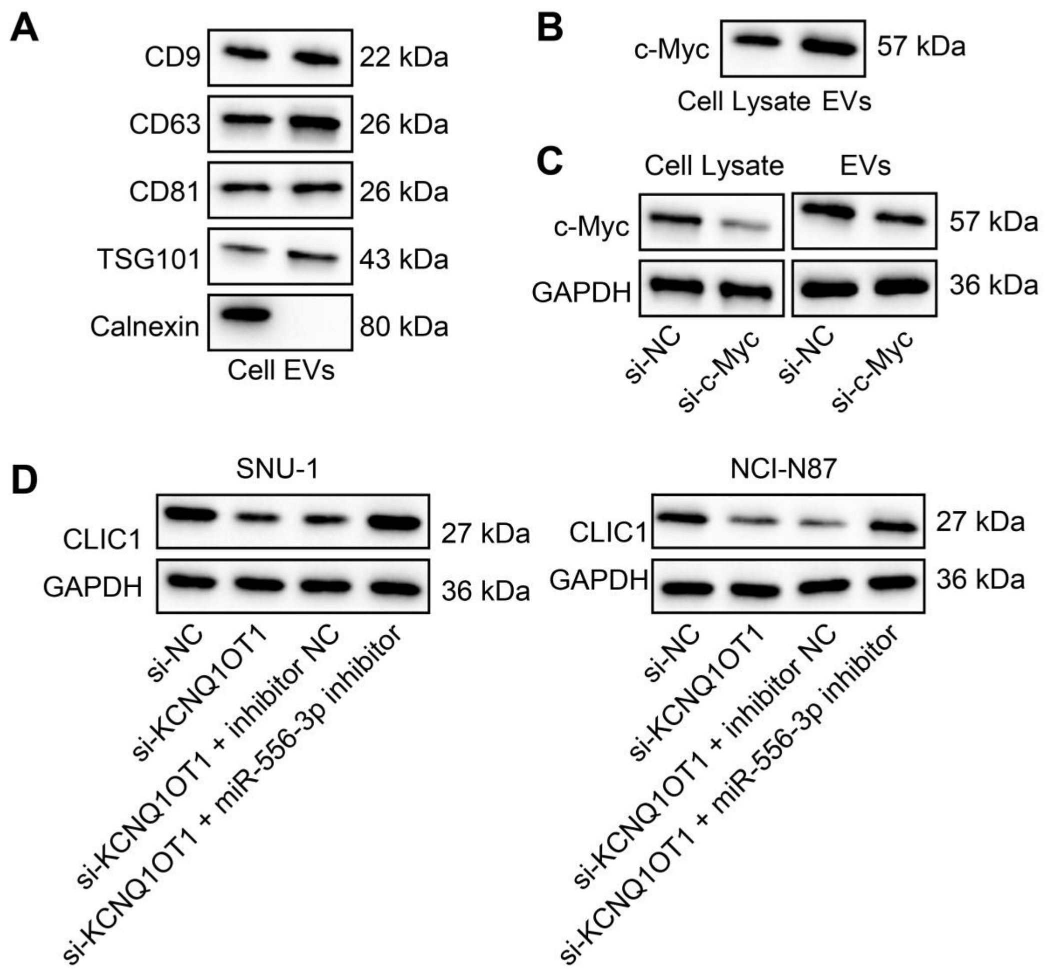

Supplementary Fig. 2 Original western blots. A, The expression of CD9, CD63, CD81, Calnexin, and TSG101 in GC cells and EVs detected by Western blot analysis. B, The protein expression of c-Myc in GC cells and EVs determined by Western blot analysis. C, Western blot analysis was used to detect the protein expression of c-Myc in OCUM-1 cells and EVs after knockdown of c-Myc. D, Western blot was used to detect the protein expression of CLIC1 after si-KCNQ1OT1 and miR-556-3p inhibitor treatment.

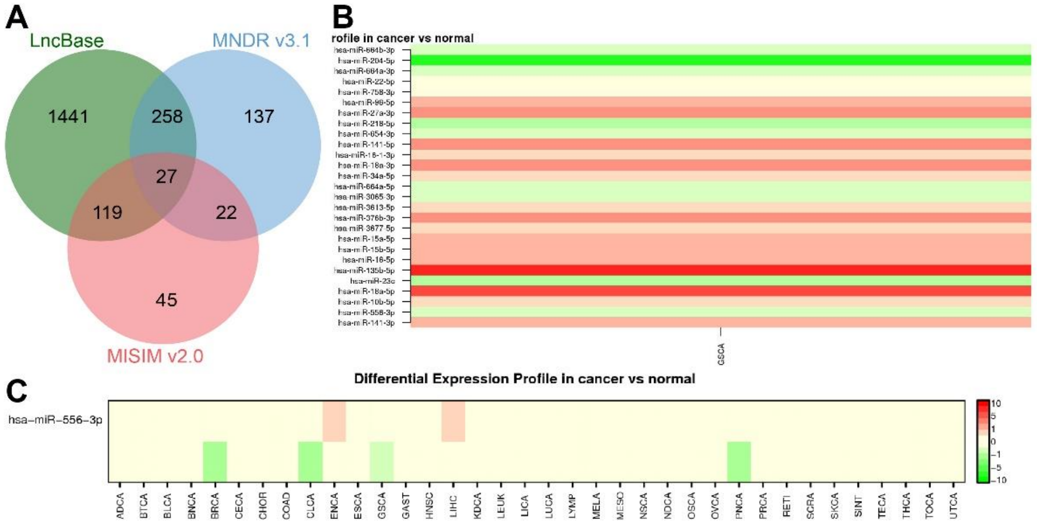

Supplementary Fig. 3 Bioinformatics analysis predicts miRNAs that lncRNA KCNQ1OT1 might bind to. A, Venn diagram of miRNAs binding to lncRNA KCNQ1OT1 predicted by the LncBase database, MNDR v3.1 (http://www.rna-society.org/mndr/) and MISIM v2.0 (http://www.lirmed.com/misim/Home). B, Expression profile of candidate miRNAs in GC (The green to red color scale indicates the log2FC value from small to large). C, Expression profile of miR-556-3p in multiple tumors.

Supplementary Table 1 Cell grouping and plasmid transfection

Group name	name
Flag-c-Myc-EVs	EVs with Flag tag and overexpression of c-Myc
si-NC	Negative control of si-RNA interference
si-c-Myc	Knockdown of CLIC1
si-KCNQ1OT1	Knockdown of KCNQ1OT1
oe-NC	Negative control of overexpression
oe-KCNQ1OT1	Overexpression of KCNQ1OT1
oe-CLIC1	Overexpression of CLIC1
si-NC	Negative control of siRNA
si-KCNQ1OT1	Knockdown of KCNQ1OT1
Inhibitor NC	Negative control of miRNA knockdown
miR-556-3p inhibitor	Knockdown of miR-556-3p
miR-NC	Negative control of overexpression of miRNA
miR-556-3p mimic	Overexpression of miR-556-3p

Supplementary Table 2 Primer sequences of RT-qPCR

Gene	Sequence
LncRNA KCNQ1OT1-F	5′-ACTCACTCACTCACTCACT-3′
LncRNA KCNQ1OT1-R	5′-CTGGCTCCTTCTATCACATT-3′
miR-556-3p-F	5′-ATATTACCATTAGCTCATCTTT-3′
miR-556-3p-R	Universal primer
CLIC1-F	5′-AGTTTTTGGATGGCAACGAGC-3′
CLIC1-R	5′-CTGGACAGGTGGAAGCGAAT-3′
c-Myc-F	5′-GGCTCCTGGCAAAAGGTCA-3′
c-Myc-R	5′-CTGCGTAGTTGTGCTGATGT-3′
GAPDH-F	5′-TGTTCGTCATGGGTGTGAAC-3′
GAPDH-R	5′-ATGGCATGGACTGTGGTCAT-3′
U6-F	5'-GCTTCGGCAGCACATATACTAAAAT-3'
U6-R	5'-CGCTTCAGAATTTGCGTGTCAT-3'
